# Making Diet Management Easier: The Effects of Nudge-Based Dietary Education and Tableware in Individuals with Both T2DM and Overweight/Obesity: A 2 × 2 Cluster Randomized Controlled Trial

**DOI:** 10.3390/nu17091574

**Published:** 2025-05-03

**Authors:** Tianxue Long, Yating Zhang, Yiyun Zhang, Yi Wu, Jing Huang, Hua Jiang, Dan Luo, Xue Cai, Rongsong Tang, Dan Zhang, Lang Peng, Xiaojing Guo, Mingzi Li

**Affiliations:** 1 School of Nursing, Peking University, Beijing 100891, China; longtianxue@pku.edu.cn (T.L.); 2011210160@bjmu.edu.cn (Y.Z.); 2211210130@stu.pku.edu.cn (Y.Z.); jiyuanwuyi@alumni.pku.edu.cn (Y.W.); 2211110257@stu.pku.edu.cn (J.H.); jianghua@bjmu.edu.cn (H.J.); luodan@njucm.edu.cn (D.L.); kameyuki@126.com (X.C.); trs1994@bjmu.edu.cn (R.T.); 2311210138@stu.pku.edu.cn (D.Z.); smallbridge@pku.edu.cn (L.P.); 2411110245@stu.pku.edu.cn (X.G.); 2 School of Nursing, Nanjing University of Chinese Medicine, Nanjing 210023, China

**Keywords:** nudge, type 2 diabetes, overweight, obesity, dietary behaviour, dietary education, tableware, dual-process model, metabolic control, psychological health

## Abstract

Background/Objectives: Traditional diet management for type 2 diabetes (T2DM) is often complex and effortful to sustain. Nudging offers low-effort and automatic approaches to dietary behaviour change yet remains underexplored in T2DM. This study evaluated the independent and combined 6-month effects of nudging education (NE) and nudging tableware (NT) on HbA1c, along with other secondary health outcomes, among adults with T2DM and overweight/obesity, compared to their non-nudge counterparts (control education, CE; control tableware, CT). Methods: A 2 × 2 factorial cluster RCT was conducted in 12 primary healthcare settings in China (pre-registered as ChiCtr2100044471). Participants were randomly assigned to the nudging education group (NE + CT), the nudging tableware group (CE + NT), the combined group (NE + NT) or the full-control group (CE + CT) for 1 month. The primary outcome was HbA1c. Secondary outcomes included dietary behaviours, metabolic indicators, and psychological health. Generalized linear mixed models were used for analysis. Results: A total of 284 participants (mean age, 52.28 years; 54.3% male) were randomly assigned and included in the analysis. After 6 months, NE and NT independently led to HbA1c reductions (−0.76%, *p* < 0.001; −0.33%, *p* = 0.042, vs. controls), with an additive but non-interactive effect when combined, resulting in a 1.04% reduction (*p* < 0.001) in the combined group. They also improved total calorie, macronutrient, and vegetable intake, FBG, plasma lipids, and BMI. NE additionally reduced diabetes distress and enhanced self-efficacy. Conclusions: Both NE and NT improved dietary and metabolic outcomes without increasing the psychological burden. The combined group showed the greatest benefits. Findings highlighted the importance of considering automatic processes in diabetes management.

## 1. Introduction

Type 2 diabetes (T2DM) affects approximately 537 million adults globally, with over 60% classified as overweight or obese, significantly increasing the risk of complications and mortality [1,2,3]. Effective metabolic control, particularly glycemic control, is crucial for delaying disease progression. Unfortunately, less than 50% of individuals achieved the goal of HbA1c < 7%, and the concurrent attainment rate with other metabolic indicators was even lower [4].

Lifestyle modifications, especially diet management, are essential for achieving these goals [4,5]. Nutritional therapy guidelines consistently recommend reducing total calorie intake, increasing vegetable consumption, and optimizing the energy supply of macronutrients [4,5,6]. Despite these well-established recommendations, their effects were pervasively plagued by low adherence and negative emotions in the real-world transformation [7,8]. More than half of the patients failed to persistently maintain a healthy diet [6,9]. Over one-third of the patients experienced psychological burdens, such as diabetes distress [10], which may further impede effective diet management.

A possible limitation of traditional diet management strategies is their intensive and cognitive resource-demanding nature, which is too complex and overwhelming for patients to follow [9,11,12]. They typically provide extensive nutritional knowledge and numeric dietary advice, and teach them to use calibrated portion control tools, requiring patients to engage in precise nutrient calculation and exert effortful self-control [13,14,15]. These strategies assume that behaviour change is predominantly driven by a rational and deliberative process, while based on the dual-process model, most dietary decisions are naturally irrational, automatic, and instinctive processes [16,17,18]. Factors such as an “obesogenic environment”, strong unhealthy habits, reward-driven impulses, and emotional influences often lead individuals to deviate from rational decision-making [19,20,21]. Relying on a deliberative approach, traditional strategies would deplete the cognitive resources and willpower of individuals when counteracting the automatic process, leading to noncompliance and frustrations [8,22,23]. It was particularly troublesome among adult patients, who manage occupational burdens and family responsibilities [11,24,25]. To address this problem, strategies to make diet management easier are needed for sustainable behaviour change, which are designed to align with the automatic decision-making process and require minimal cognitive effort.

In contrast to the complex and deliberative approach, nudging—proposed by Thaler and Sunstein in 2008—refers to modifying choice architecture to influence behaviour predictably, without forbidding any options [20,26,27]. Nudging works through an automatic process by activating related associative mental or motor responses, making healthy options easier to choose with fewer cognitive resources and less psychological burden [20,28,29]. In recent years, nudging has emerged in public health and nutrition [20,30]. For instance, changing products’ placement and serving sizes can encourage participants to consume more fruits and vegetables and reduce unhealthy snacks [28]. Applying simple, salient, and healthy goal-priming prompts on the menu or in the surroundings promotes healthy food selections [20,31]. These trials present an expectant prospect for nudging in diet management.

Nudge strategies have also been applied in chronic disease management, with particular success in medications, physical activity, and weight control [32,33,34]. However, effective and high-quality evidence on sustainable disease control and diet management, especially for diabetes, remains sparse [32,33]. Existing studies incorporated nudge-based strategies in interventions such as text reminders and social norms, yet they failed to address some essential components of diet management, including the content of nutritional guidance and behavioural practice [32,33,35]. Furthermore, effective nudge strategies for modifying eating behaviour, such as simplification, default, salience, or priming, have not been fully integrated into diabetes diet management [20,26,30,35]. Additionally, many successful nudging interventions have often occurred in nudge-designed public settings, limiting their applicability to real-life individual decision-making environments [20]. Despite growing interest in nudging, its application in diet management for T2DM remains underexplored.

Since diet management is primarily delivered through education and supportive tools, transforming them into nudge-based alternatives could offer promising solutions [7,13,14,15,35]. Preliminary findings from our nudge-based education program showed significant improvements in metabolic control and dietary behaviour among adults with T2DM and overweight/obesity over 3 months in a limited sample [36]. Moreover, our previous study indicated that a set of nudging tableware successfully promoted healthier food choices among young adults during a single meal [37]. It was unclear whether these interventions would remain effective over a longer period in a larger population of T2DM individuals and how they would perform when combined.

Thus, this study aimed to examine the individual and combined effects of the nudge-based dietary education and nudging tableware on HbA1C as the primary outcome, as well as on the secondary outcomes, including dietary behaviours, other metabolic indicators, and psychological health, among adults with type 2 diabetes and overweight/obesity over 6 months by a large cluster-randomized controlled trial.

## 2. Materials and Methods

### 2.1. Participants

Individuals with type 2 diabetes and overweight/obesity were eligible. The specific eligibility criteria for research centres and individuals were as follows:

(1) Eligibility for research centre

Inclusion criteria: at least 100 eligible patients registered and receiving stable healthcare services; at least five healthcare professionals; sufficient space for group classes of 8–10 people; with a similar scale and economic condition; a qualified clinical testing centre capable of conducting the measurements.

Exclusion criteria: involved in other research projects or unwilling to cooperate.

(2) Eligibility for individual subjects

Inclusion criteria: patients diagnosed with type 2 diabetes, aged 18–59 years, having a body mass index (BMI) ≥24 kg/m^2^, and living in Beijing for the next two years. Participants were asked to report their intention to reside in Beijing for the next two years during the informed consent process. Participants who relocated during follow-up were treated as lost to follow-up.

Exclusion criteria: advanced complications of diabetes (proliferative retinopathy, nephropathy stage IV or above, creatinine levels ≥2 mg/dL, heart function below grade III, cerebrovascular accident sequelae, and diabetic foot above grade I); patients currently receiving injectable hypoglycemic medications, as their dietary requirements differ significantly and were not addressed in this study; patients who experienced a psychiatric or cognitive impairment that may hinder cooperation (schizophrenia, dementia, major depression, and substance abuse); or patients who had participated in other research projects.

### 2.2. Study Design, Randomization, and Masking

This study was a clustered randomized controlled trial with a 2 × 2 factorial design, conducted across 12 suburban primary healthcare settings in Beijing, China. Cluster randomization was selected to minimize contamination from patient or physician preferences. A factorial design was selected to explore both the main and interaction effects, allowing a comprehensive evaluation of the nudge-based education and nudging tableware set and identifying the optimal clinical applications. Study procedures were designed and reported following the CONSORT consensus for clustered randomized controlled trials [25]. Ethical approval was obtained from the Peking University Institutional Review Board (IRB00001052-21031). The trial was previously registered at the Chinese Clinical Trial Registry (ChiCtr2100044471). All participants signed informed consent forms.

This study included two types of diet management (dietary education and tableware), each with two treatment levels—either with or without nudge strategies (intervention vs. control). Specifically, referring to dietary education, the intervention was a nudge-based dietary education program (nudging education, NE), whose control was traditional dietary education (control education, CE). Referring to the tableware, the intervention was the use of the nudging tableware set (nudging tableware, NT), whose control was the use of usual household tableware (control tableware, CT).

**Figure 1:**
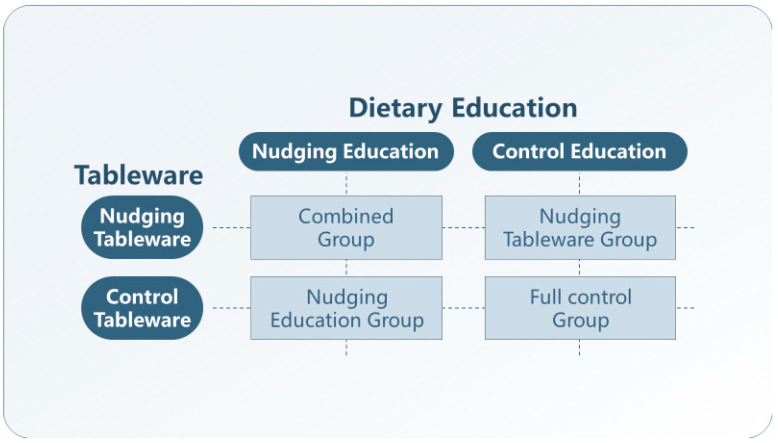
The illustration of treatments group based on a 2 × 2 factorial design.

A researcher uninvolved in the trial used computer-generated block randomization via SPSS software and allocated research centres to one of the four groups in a 1:1:1:1 ratio. The 4 groups were as follows (see Figure 1): (a) the nudging education group, receiving NE and CE treatments; (b) the nudging tableware group, receiving CE and NT treatments; (c) the combined group, receiving NE and NT treatments; (d) the full-control group, receiving CE and CT treatments.

Allocation was concealed by the central research centre. The results were kept unknown to the staff and participants at each site.

### 2.3. Treatments

All treatments in this study shared the same content, goal, and duration: they adopted a recommended balanced calorie-restricted diet according to the Chinese Guidelines for the Prevention and Treatment of T2DM, and the treatments were carried out for a month. For the interventions, we designed 2 types of nudge-based diet management (nudging education and nudging tableware), whose nudge elements were selected from 3 acknowledged practicing frameworks: MINDSPACE, TIPPME, and Sunstein’s list of the most important nudges [12,26,28,38]. In contrast, for their non-nudge controls, traditional diet management (control education and control tableware) using deliberative approaches was adopted. All interventionists were local primary healthcare providers, and those who received standard training and passed the assessment were eligible to conduct the study.

#### 2.3.1. Nudging Education (NE)

This was a nurse-led, face-to-face, and structured education program. It was conducted weekly in 45 min sessions over four weeks with a group of 8–10 participants. All sessions and teaching materials were designed by nudge principles to meet the needs of various aspects of diabetes diet management, primarily focusing on simplification, availability, warning, pre-commitment, and reminders, with additional strategies such as salience, priming, and effects (detailed in Supplementary Table S1, and see [36]). It was conducted with low-literacy and visual teaching materials, using life-sized food cards (100 kcal/piece, except vegetables, which were considered to have no calories), “traffic light” classification cards, and illustrated wall charts. In principle, the complex nutritional information and healthy eating practices were transformed into simple and practical rules, such as using “traffic light” classification with emotional arousal signs, the strategic placement of illustrations, and a one-sentence “golden rule”. Additionally, individualized diet adjustment instruction was provided to help them reduce calorie intake using a visualized “substitution rule” instead of precise calculation.

#### 2.3.2. Nudging Tableware (NT)

**Figure 2:**
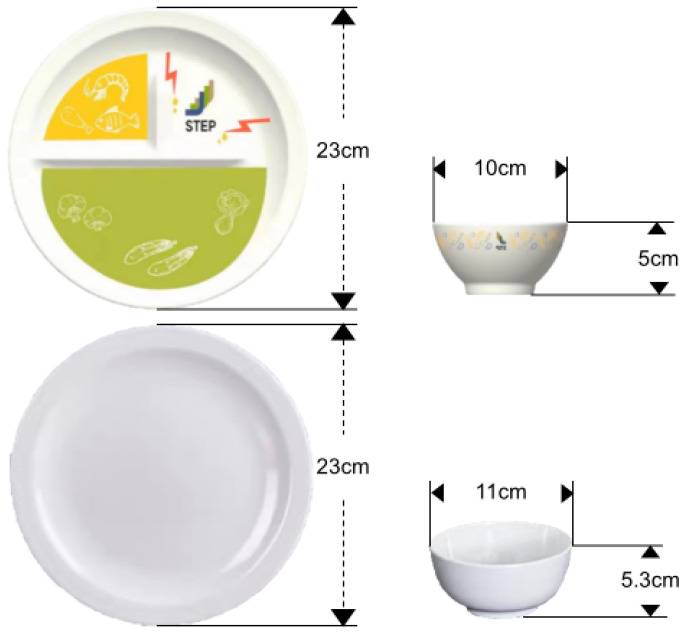
The illustration of the nudging tableware set.

Participants used a set of nudging tableware under this treatment, which contained a bowl and a plate (Figure 2, and see [35]). The design incorporated nudge strategies, including default, size, functionality, information, simplified priming, salience, and warning visual cues, to guide healthier choices.

The nudging plate was designed with half of a portion for vegetables with a green background and vegetable patterns, a quarter for protein with an orange background and high-quality protein patterns, and a quarter with oil droplets and warning lightning patterns. Each area was separated by protruding arrises. Notably, the oil drop drainage area was slightly lower than the other two segments to gather excess sauce from the dishes. The nudging bowl was painted with various grain patterns externally, and a protruding marker line was set inside, indicating “stop taking”. The nudging tableware set had a reduced capacity while maintaining the same visual perception of usual-size tableware due to the thick rims and greater slope of the side, subtly limiting food intake.

Participants were taught how to use the tableware by nurses and took home a low-literacy paper guide and a video manual. They were required to use it at least once per day for a month and upload their meal photos. Nurses provided personal advice on the placement of the dishes to assist them in using the tableware without explicit and specific nutritional instruction (e.g., “the green section has not been filled” or “Avoid placing food in the oil droplet area”). Their food intake was unrestricted, with participants being allowed to choose and retake freely.

#### 2.3.3. Control Education (CE)

Traditional dietary education was conducted as the control education. It shared the same educational contents as NE but emphasized accurate calculation and strict control, such as providing numeric nutrition information, teaching patients to accurately calculate and control calorie and macronutrient intake by measuring food weight, checking food labels, using food scales and measuring jugs, and implementing a food exchange strategy (detailed in Supplementary Table S1). They were trained to apply diet control using the following procedures: (1) Calculating total daily calorie intake in three steps: (a) calculating standard body weight; (b) self-assessing physical activity level, which was adjusted for age and BMI; (c) calculating daily calorie requirements based on standard body weight and physical activity level. (2) Calculating daily intake (g/d) of grains, vegetables, fruits, animal food, milk products, and cooking oil based on total calorie intake and recommended balanced macronutrient composition. (3) Dividing the above food intake into three meals and extra meals. Likewise, it was also nurse-led, face-to-face, group-based, once a week for 45 min, with 8–10 participants, using the standard Diabetes Management Education and Support (DMES) materials published by the Chinese Diabetes Society.

#### 2.3.4. Control Tableware (CE)

Usual household tableware was set as the control tableware treatment, with participants making conscious decisions about food types and serving sizes based on their own knowledge and self-control efforts during meals.

### 2.4. Outcomes and Data Collection

Data were collected at baseline and three and six months post-intervention. The primary outcome was HbA1c. Secondary outcome measures included dietary behaviours (total energy intake, macronutrient composition, and vegetable intake), other metabolic indicators (fasting blood glucose (FBG), BMI, total cholesterol (TC), triglycerides (TGs), low-density lipoprotein cholesterol (LDL), and high-density lipoprotein cholesterol (HDL)), and psychological health (diabetes distress and diabetes management self-efficacy).

Fasting (>8 h) blood samples were collected via venipuncture and analyzed at the centralized laboratory centre. Weight and height were measured while wearing light clothing and no shoes, with calibrated digital scales to the nearest 0.1. All questionnaires were self-reported following the unified instructions of trained researchers, including a 48 h retrospective dietary record for dietary behaviours, and the Diabetes Distress Scale and the Diabetes Management Self-Efficacy Scale for psychological health. Demographic data, including age, gender, and disease duration, were retrieved from electronic medical records when participants were enrolled.

### 2.5. Statistical Analysis

According to our preliminary analysis and previous studies on diet education for T2DM [14,36,39], we calculated that a sample size of 268 participants would provide 80% power to detect a 0.5% significant difference in HbA1c (SD 0.7%) between intervention and control at a significance level of 0.05, assuming an ICC of 0.02, a potential cluster size of 100, and a 20% predicted dropout rate [40].

Generalized linear mixed models (GLMMs) were used to assess the intervention effects of nudge-based diet management from baseline to 6-month follow-up. Data from the 3-month follow-up were also included in the models to assist estimation. GLMMs included dummy variables for time (reference: baseline), dietary education (reference: control education), and tableware (reference: control tableware) as fixed effects, along with their 2-way and 3-way interactions. Clusters were treated as second-level random factors, and individuals were treated as first-level random factors in the models. The intraclass correlation coefficient (ICC) of the primary outcome, HbA1c, was calculated based on an empty model using a one-way random effects structure, corresponding to the ICC (1). The 2-way interactions of dietary education or tableware with time assessed their independent effects, indicating differences in changes for outcomes over time between each nudge-based intervention (nudging education or nudging tableware) and its respective non-nudge control. The 3-way interactions assessed their combined effects on outcomes, identifying potential synergistic or antagonistic interactions. If the 3-way interaction (dietary education × tableware × time) was non-significant (*p* > 0.05), it was excluded, and independent effects were re-assessed. Models were adjusted for variables, including age, sex, education, family income, and baseline. An intention-to-treat (ITT) analysis was used to incorporate the loss of follow-up data. Missing values were addressed using multiple imputations, based on the assumption of random missing data. Statistical analysis was performed using IBM SPSS Statistics, Version 24.0.

## 3. Results

### 3.1. Participants and Baseline Characteristics

**Figure 3:**
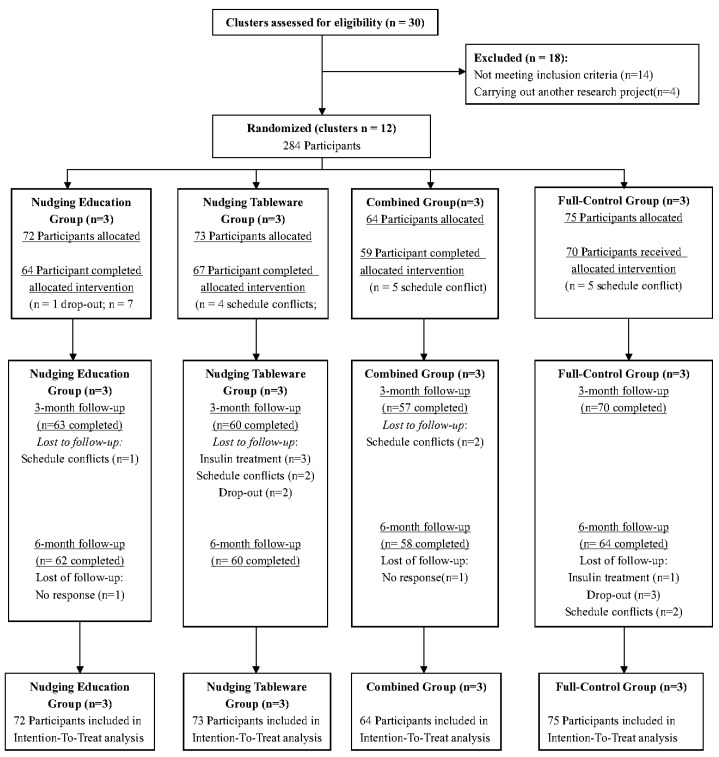
Study participants flowchart.

Twelve eligible research centres enrolled 284 participants from July 2021 to September 2022. The primary reasons for the loss to follow-up were scheduling conflicts, receiving insulin treatment, and non-responsiveness. A detailed participant flowchart is provided in Figure 3. During the intervention period, 119 out of 136 participants who received nudging education completed the treatment, and 114 out of 137 participants who received nudging tableware were adherent for ≥70% of the time.

In the ITT samples, the mean age (SD) was 52.28 (8.02) years, and 54.3% were males. The HbA1c level was 7.62% (1.57%) at the baseline. More details are summarized in Table 1. The baseline characteristics were comparable among patients regardless of intervention assignment or follow-up completion. The ICC for HbA1c was 0.003, indicating minimal clustering across centres.

### 3.2. Effects of Nudge-Based Diet Management

The six-month effects are presented in Table 2. The three-month effects, estimated means for outcomes, and pairwise comparisons of each group at various time points are provided in Supplementary Tables S2–S4.

#### 3.2.1. Primary Outcome

At 6 months, the main effect of time, which indicated a change for the full-control condition, exhibited no significant change in HbA1c levels (−0.10%, 95% CI: −0.36 to 0.16). Nudge education (NE) showed a significantly lower HbA1c compared to control education (CE) over time (−0.76%, 95% CI: −1.08 to −0.45). Nudging tableware (NT) showed a significantly lower HbA1c compared to control tableware (CT) (−0.33%, 95% CI: −0.64 to −0.01). No significant 3-way interaction effect was observed (F = 1.826, *p* = 0.124). The reduction was greatest in the combined intervention group (−1.04%, 95% CI: −1.36, −0.73) (Table 1). The six-month effect of NE was greater than the three-month effect (mean difference = −0.27%, *p* = 0.002).

#### 3.2.2. Dietary Behaviours

As the main effect of time, the total calorie intake increased significantly (90.51 kcal/d, 95% CI:10.47 to 170.55). Compared to their controls, NE significantly reduced the intake of participants by 291.90 kcal/d, and NT reduced their intake by 454.59 kcal/d. With the increased 3-way interaction effect, participants in nudging education, nudging tableware, and combined groups reduced their total calorie intake by 173.08, 335.77, and 354.23 kcal/d compared to the baseline, respectively.

The interventions resulted in a decrease of most of the macronutrient intake, including carbohydrates, proteins, and lipids (Table 2).

For the vegetable intake, there was no significant main effect of time. Compared to their control, NE significantly increased the intake of participants by 112.24 g/d, and NT increased it by 119.20 g/d. With the slightly decreasing 3-way interaction effect, participants in the nudging education, nudging tableware, and combined groups significantly increased their vegetable intake by 115.21, 112.17, and 146.41 over 6 months, respectively.

#### 3.2.3. Secondary Metabolic Indicators

##### Fasting Blood Glucose (FBG)

No significant main effect of time was observed in FBG (mmol/L) (−0.01, 95% CI: −0.46 to 0.44). Compared to their controls, the reduction contributed by NE was 0.62 (95% CI: −1.15 to −0.08), and the reduction by NT was 0.98 (95% CI: −1.52 to −0.45). There was no significant interaction effect (F = 0.021, *p* = 0.890).

##### Plasma Lipids

TC (mmol/L) was significantly increased as the main effect of time (0.38, 95% CI: 0.10 to 0.65). A significant 3-way interaction effect was observed (0.63, 95% CI: 0.05 to 1.21). Compared to their controls, the reduction contributed by NE was 0.71 (95% CI: −1.11 to −0.31), and the reduction contributed by NT was 0.69 (95% CI: −1.09 to −0.30) over time. The changes for nudging education, nudging tableware, combined, and the full-control groups were −0.33, −0.32, −0.40, and +0.38 mmol/L, respectively.

There was no significant main effect of time in TG (mmol/L). Only the NT contributed to the estimated reduction of −0.66 (95% CI: −1.17 to −0.15) compared with the CT.

LDL (mmol/L) analysis revealed no significant main effect of time. Only the NT contributed to the estimated reduction of −0.36 (95% CI: −0.59 to −0.14) compared with the CT.

HDL (mmol/L) was significantly increased over time (0.21, 95% CI: 0.05 to 0.37). No other significant effect was observed.

##### Body Mass Index (BMI)

There was no significant main effect of time regarding the BMI. The NE contributed to the reduction of 0.30 kg/m^2^ (95% CI: −0.70 to −0.09) compared to CE, and NT contributed to the reduction of 0.61 kg/m^2^ (95% CI: −1.00 to −0.21) compared to CT.

#### 3.2.4. Psychological Health

Diabetes distress was decreased by 2.50 points (95% CI: −4.79 to −0.20) over 6 months for the main effect of time. Compared to the controls, NE further reduced distress by 3.95 points (95% CI: −6.69 to −1.20), whereas NT had no significant effect. Subscale analyses revealed that NE significantly decreased emotional burden and regime-related distress, and NT demonstrated a significant decrease in interpersonal distress and emotional burden (Table 2). The six-month effect of NE was greater than the three-month effect (Supplementary Table S4).

A 0.05-point (95% CI: 0.02 to 0.09) increase in diabetes self-management efficacy was observed for the main effect of time. NE led to a significant increase (0.05, 95% CI: 0.01 to 0.09) compared to its control, while NT exhibited no effect. Subscale analyses (Table 2) indicated that NE primarily improved self-efficacy in diet and blood sugar, and drug adherence.

## 4. Discussion

To the best of our knowledge, this is the first large-scale randomized controlled trial to comprehensively assess a multi-strategy nudging approach in diabetes dietary management. Both 1-month of nudging education and nudging tableware improved dietary behaviour and metabolic control without inducing negative emotional responses in adults with type 2 diabetes and overweight/obesity over 6 months. For most of the outcomes, combining them proved to be the most effective. The effects on HbA1C and diabetes distress were more pronounced compared to three months.

These results were consistent with or even better than many previous dietary interventions, which primarily involved traditional medical nutrition therapy and health education approaches without the use of nudge strategies [39,41]. More encouragingly, our nudge interventions outperformed several existing nudge-based interventions for diabetes management, particularly in improving actual dietary behaviour and metabolic control, areas where many nudging diet interventions for diabetes have shown limited success [32]. It may be because our study extended previous approaches, which primarily modified the delivery mode of dietary programs using nudge strategies, such as social norms and reminders, which still require active cognitive engagement in dietary practice as usual [7,32,35]. By integrating nudge strategies into educational content and daily food choice contexts, we provide more intuitive and accessible options for dietary practice. The multi-strategy nudge design also allowed complementary and interactive effects between different nudge elements, such as using affective signs on simple and salient prompts [12,38]. Our solution also fills the gap in nudge-based diet management for T2DM by fostering personalized dietary changes instead of relying on nudge-designed public settings. Additionally, we accommodated the intervention to local contexts by simplifying resource requirements for interveners and incorporating local dietary culture. Delivered by trained local primary healthcare, our intervention sustained its effectiveness for 6 months, able to meet national biannual health education requirements without an extra workload while maintaining high fidelity, acceptability, and sustainability [37,42]. This makes it well suited for widespread implementation, particularly in resource-limited areas. Moreover, recent findings suggest that the COVID-19 pandemic has had a disruptive impact on eating behaviours among individuals requiring dietary management, highlighting the need for supportive and sustainable behavioural strategies in response to similar future public health crises [43]. Conducted during the pandemic period, our study emphasized the potential efficacy of our low-burden, nudge-based dietary interventions. It would be valuable for future research to assess their adaptability across various populations and broader global contexts.

Improving metabolic control, particularly glycemic control, is the primary target for T2DM management. Without significant changes in medication use or physical activity levels, both nudging interventions resulted in a reduction in HbA1c over six months. It outperformed several interventions primarily targeting deliberative decision-making processes [14,44]. Our nudging education exhibited sustained benefits comparable to some resource-intensive and long-term nutritional programs [39]. Nudging tableware also yielded significant improvements in HbA1c, fasting blood glucose, plasma lipid profiles, and BMI, with relatively lower intervention intensity. Previous studies on portion-control tableware have reported only minor and short-term effects in T2DM [15,45]. The combined approach obtained the greatest effect, likely due to an additive rather than interactive mechanism, suggesting a potential metabolic ceiling effect. Given the importance of metabolic control in delaying T2DM progression, we anticipate that our nudge-based diet management, particularly with a combined approach, can contribute to reduced diabetes-related complications and mortality [14]. Future cost-effectiveness analyses are warranted to determine the optimal implementation model from the practical aspect.

These sustained improvements can be attributed to reductions in total calorie and macronutrient intake. The automatic cueing mechanism of nudging, which involves the associative activation of habitual responses, likely played a role [20,28,29]. Our nudging education would reshape how participants acquire nutritional knowledge and carry on diet practice, shifting new, complex, and high-literacy learning to familiar, simple, and intuitive formats such as “equal-size food card”, “traffic light classification”, and “golden rules”. These strategies guide patients to consume more vegetables and reduce high-lipid food intake with minimal cognitive effort. By leveraging heuristics and emotional arousal, participants may have relied on automatic internal cues, such as the “red for stop” mental shortcut or emotional reinforcement, when making food choices [29]. Referring to our nudging tableware, it provided external cues as healthier “default options” to automatically manipulate diet, such as reduced portion sizes with equal visual perception, separate sections with protruding arrises, and an oil drainage area. Visual prompts on the tableware would also potentially prime participants to select healthier foods and avoid excessive fat consumption. Additionally, from a homeostasis perspective, increased vegetable intake mitigated blood sugar fluctuations, thereby reducing food cravings [16,46]. In summary, both nudging interventions bypassed the barriers to remembering complex nutritional information and executing effortful dietary practices. In contrast, traditional strategies of diet management often rely on rules-based and rational judgement, inhibitory control, and other deliberative processes, such as specific and numerical nutritional knowledge and strict self-control, which may lead to an intention–behaviour gap [17]. Moreover, when individuals experience a conflict between health beliefs and behavioural impulses, they often adjust their beliefs rather than their behaviours to feel better, leading to failure in adhering to a healthy diet [12].

The effects of nudging education and tableware on psychological health were different. Nudging education reduced the diabetes distress of participants, especially emotional burden and regimen-related distress, and enhanced the diet-related management efficacy. The nudging tableware alleviated emotional burden and interpersonal distress. It may suggest that they influence different psychological mechanisms in the automatic process. Based on the intervention design, nudging education enhanced self-efficacy sources and fostered a positive attitude toward healthy eating by providing effective signs [44]. As classified by Sunstein, it can be regarded as an “educative nudge” that empowers individuals by augmenting knowledge and capacities [47]. Nudging tableware facilitated individual meal serving without disturbing communal eating traditions, potentially obtaining greater interpersonal support. Based on choice transparency, our nudging education may be classified as a transparent nudge, allowing participants the conscious intention to engage in decision-making [48]. In contrast, the nudging tableware may function as a non-transparent nudge, with little perception of psychological change [48]. This is based on the perspective that explicit self-reported outcomes are typically shaped by deliberative thinking [17]. From the cognitive neuroscience standpoint of the automatic process, education may alter the areas of affects and self-representativeness as an internal cue is activated, whereas tableware may change the area of sensory processing as an external cue is activated [49]. Further investigations are required to explore the mechanisms underlying behaviour change caused by different types of dietary nudges and to advance the dual-process model for health behaviour regulation across multiple levels.

The strengths of this study include its theory-based design, multi-strategy nudge approach, different types of diet management considerations, simple practice method, shorter intervention period, more effective and comprehensive range of results, and real-life clinical settings. However, several limitations should be acknowledged. First, most participants were middle-aged and from northern China. More evidence from younger participants and different regions is needed. Second, physiological appetite regulation mechanisms for diabetes, such as insulin resistance and abnormal glycemic responses, may influence automatic nudge cueing responses. Given the comparable metabolic status across groups at baseline, we believe these effects were likely balanced. Third, interobserver variability and self-reported measures could introduce bias. We adopted strict quality control to minimize bias across research centres, such as unifying measuring standards and scales, training researchers, and conducting process evaluations. Fourth, the applicability of our nudging tableware was limited to some extent, primarily suitable for rice-based dining meals, and ignoring fruits and snacks as extra meals, necessitating the further development of nudging tools. Furthermore, to advance the theoretical foundations of the dual-process model and guide practical applications, the specific decision-making processes underlying different types of nudges require further investigation across behavioural science disciplines, such as cognitive neuroscience, to clarify the mechanisms of behaviour change.

## 5. Conclusions

In conclusion, our nudging dietary education and nudging tableware significantly improved metabolic control, particularly glycemic control, and dietary behaviour in adults with T2DM and overweight/obesity over 6 months, compared to traditional diet management. When combined, it was more effective. The nudging education also demonstrated advantages in reducing diabetes distress and enhancing self-efficacy. These findings provide a simple and acceptable solution for diet management in primary healthcare settings. Future research should explore broader application scenarios, including diverse nudging tools and integration with other diabetes management interventions, conduct economic evaluations to inform real-world implementation, and further advance the theoretical investigation of the dual-process model for health behaviour.

## Figures and Tables

**Table 1:** Baseline characteristics of participants.

Variables	Total (N = 284)	Nudging Education Group (N= 72)	Nudging Tableware Group (N= 73)	Combined Group (N= 64)	Full-Control Group (N = 75)
Demographic					
Age (years)	52.28 (8.02)	52.75 (7.11)	53.19 (6.13)	53.53 (6.41)	49.97 (10.77)
Gender (male)	153 (54.3%)	35 (51.5%)	38 (52.1%)	29 (45.3%)	51 (66.2%)
Education					
Junior high school and below	88 (31.2%)	20 (29.4%)	18 (23.9%)	22 (34.4%)	28 (32.2%)
Senior high school	106 (37.6%)	30 (44.1%)	33 (46.5%)	19 (29.7%)	24 (30.3%)
College and above	88 (31.2%)	18 (26.5%)	22 (29.6%)	23 (35.9%)	25 (32.9%)
Family income per month (CNY)					
≤5000 (695 USD)	105 (37.23%)	24 (35.3%)	24 (32.9%)	32 (50%)	25 (32%)
>5000 (695 USD)	177 (62.76%)	44 (64.7%)	49 (67.1%)	32 (50%)	52 (68%)
Comorbidity	191 (67.7%)	41 (60.3%)	49 (67.1%)	42 (65.6%)	59 (76.6%)
Oral hypoglycemic agents	259 (91.8%)	60 (88.2%)	67 (91.8%)	57 (89.1%)	75 (97.4%)
Smoking	72 (25.9%)	16 (23.5%)	18 (25%)	15 (24.2%)	23 (30.3%)
Drinking	105 (38.3%)	28 (41.2%)	30 (42.9%)	17 (28.3%)	30 (39.5%)
Metabolic indicator					
HbA1C (%)	7.62 (1.67)	7.57 (1.59)	7.54 (1.74)	7.67 (1.64)	7.68 (1.73)
Fasting blood glocose (FBG, mmol/L)	8.45 (2.75)	8.39 (2.42)	8.90 (3.08)	8.38 (2.98)	8.16 (2.47)
Total cholesterol (TC, mmol/L)	4.72 (1.16)	4.69 (1.25)	4.94 (1.13)	4.77 (1.09)	4.49 (1.13)
Triglyceride (TG, mmol/L)	2.07 (1.78)	1.76 (0.94)	2.54 (2.88)	1.89 (1.08)	2.06 (1.31)
Low density lipoprotein (LDL, mg/dL)	3.05 (0.97)	2.70 (0.89)	3.31 (0.98)	3.15 (0.97)	3.04 (0.95)
High density lipoprotein (HDL, mg/dL)	1.22 (0.56)	1.23 (0.33)	1.28 (0.85)	1.22 (0.45)	1.15 (0.42)
Body mass index (BMI, kg/m^2^)	27.77 (3.70)	27.63 (2.91)	28.25 (4.56)	27.33 (3.01)	27.80 (3.92)
Dietary Behaviour					
Total calorie intake (kcal/d)	1660.22 (416.58)	1705.85 (433.06)	1620.12 (434.72)	1695.72 (503.85)	1638.88 (324.14)
Carbohydrate intake (g/d)	216.04 (59.12)	226.02 (61.03)	201.68 (48.88)	224.60 (73.24)	214.04 (52.42)
Protein intake (g/d)	65.95 (18.21)	68.41 (19.43)	63.13 (20.49)	65.24 (20.71)	66.14 (13.46)
Lipid intake (g/d)	50.03 (19.92)	49.00 (24.39)	50.76 (19.55)	50.86 (19.30)	49.92 (16.44)
Vegetable intake (g/d)	227.85 (82.20)	224.16 (101.87)	238.92 (80.86)	227.80 (80.86)	223.91 (68.01)
Psychological status					
Diabetes distress score	30.67 (12.76)	31.65 (12.82)	29.97 (12.13)	30.95 (15.38)	30.22 (10.93)
Diabetes self-management efficacy score	0.75 (0.17)	0.75 (0.15)	0.76 (1.83)	0.75 (0.20)	0.74 (0.16)

**Table 2:** The 6-month intervention effects of nudge-based diet management.

	Pairwise Contrasts to Estimated Mean Difference in Each Treatment Group (6 Months–Baseline)				Model Estimation of Intervention Effects by Time (6 Months–Baseline)			
	Nudging Education Group	Nudging Tableware Group	Combined Group	Full Control Group	Dietary Education × Time (NE vs. CE) †	Tableware × Time (NT vs. CT) †	Dietary Education × Tableware × Time	Time ‡
	Mean Difference (95% CI)				Adjusted β Coefficient (95% CI)			
Metabolic Indicators								
HbA1C (%)	−1.01 (−1.33, −0.68) *	−0.59 (−0.91, −0.29) *	−1.04 (−1.36, −0.73) *	0.06 (−0.24, 0.36)	−0.76 (−1.08, −0.45) *	−0.33 (−0.64, −0.01) *	NA	−0.10 (−0.36, 0.16)
FBG (mmol/L)	−0.92 (−1.47, −0.38) *	−1.27 (−1.81, −0.75) *	−1.29 (−1.85, −0.73) *	0.26 (−0.25, 0.77)	−0.62 (−1.15, −0.08) *	−0.98 (−1.52, −0.45) *	NA	−0.01 (−0.46, 0.44)
TC (mmol/L)	−0.33 (−0.63, −0.04) *	−0.32 (−0.60, −0.03) *	−0.40 (−0.70, −0.10) *	0.38 (0.10, 0.65) *	−0.71 (−1.11, −0.31) *	−0.69 (−1.09, −0.30) *	0.63 (0.05, 1.21) *	0.38 (0.10, 0.65) *
TG (mmol/L)	0.46 (−0.06, 0.97)	−0.55 (−1.05, −0.04) *	−0.07 (−0.60, 0.46)	0.23 (−0.25, 0.72)	0.34 (−0.16, 0.85)	−0.66 (−1.17, −0.15) *	NA	0.18 (−0.25, 0.60)
LDL (mmol/L)	−0.03 (−0.25, 0.20)	−0.28 (−0.50, −0.07) *	−0.35 (−0.58, −0.12) *	0.11 (−0.10, 0.32)	−0.10 (−0.32, 0.12)	**−0.36 (−0.58, −0.14)**	NA	0.09 (−0.09, 0.28)
HDL (mmol/L)	−0.05 (−0.24, 0.15)	−0.01 (−0.20, 0.18)	−0.01 (−0.21, 0.19)	0.28 (0.10, 0.47) *	−0.17 (−0.36, 0.02)	−0.14 (−0.33, 0.05)	NA	0.21 (0.05, 0.37) *
BMI (mmol/L)	−0.50 (−0.89, −0.10) *	−0.79 (−1.18, −0.40) *	−0.80 (−0.12, −0.39) *	−0.08 (−0.20, 0.45)	−0.30 (−0.70, −0.09) *	−0.61 (−1.00, −0.21) *	NA	−0.05 (−0.38, 0.28)
Dietary Behaviour								
Total Calories (kcal/d)	**−173.08** (−265.00, −81.16) *	**−335.77** (−434.04, −237.50) *	**−354.23** (−458.23, −250.23) *	118.82 (33.34, 204.31) *	**−291.90** (−417.43, −166.37) *	**−454.59** (−584.84, 324.34) *	273.44 (83.10, 463.78) *	118.82 (33.34, 204.31) *
Carbohydrate (g/d)	−25.40 (−40.17, 10.64) *	−44.51 (−60.29, −28.72) *	−56.74 (−73.44, −40.03) *	19.91 (6.18, 33.64) *	−45.31 (−65.48, −25.56) *	−64.42 (−85.34, 43.50) *	33.08 (2.51, 63.66) *	19.91 (6.18, 33.64) *
Protein (g/d)	−2.90 (−7.88, 2.09)	−9.24 (−14.57, −3.91) *	−9.61 (−15.24, −3.97) *	−0.44 (−5.01, 4.19)	−1.55 (−6.65, 3.56)	−7.82 (−12.96, −2.68) *	NA	−0.86 (−5.00, 3.27)
Lipid Intake (g/d)	−6.68 (−11.60, −1.76) *	−13.39 (−18.65, −8.13) *	−9.87 (−15.44, −4.31) *	4.55 (0.03, 9.13) *	−11.23 (−17.95, −4.51) *	−17.94 (−24.91, −10.97) *	14.75 (4.56, 24.93) *	4.55 (0.03, 9.13) *
Vegetable Intake (g/d)	115.21 (91.15, 139.28) *	112.17 (86.44, 137.90) *	146.41 (119.17, 173.64) *	−7.03 (−29.41, 15.36)	112.24 (89.37, 155.11) *	119.20 (85.10, 153.31) *	−88.01 (−137.85, −38.17) *	−7.03 (−29.41, 15.36)
Psychological Health								
Diabetes Distress Scale (DDS)	−6.05 (−8.81, −5.28) *	−3.09 (−5.80, −0.40) *	−6.81 (−9.66, −3.97) *	−2.80 (−3.68, −1.51) *	−3.95 (−6.69, −1.20) *	−2.07 (−4.82, 0.67)	NA	−2.50 (−4.79, −0.20) *
DDS—Emotional Burden	−2.56 (−3.45, −1.67) *	−1.50 (−2.37, −0.63) *	−1.88 (−2.80, −0.95) *	−0.13 (−0.897, 0.71)	−2.43 (−3.66, −1.20) *	−1.37 (−2.58, −0.16) *	2.05 (0.29, 3.82) *	−0.13 (−0.97, 0.71)
DDS—Physician-Related Distress	−1.60 (−2.44, −0.77) *	−1.65 (−2.47, −0.83) *	−1.52 (−2.38, −0.65) *	−0.62 (−1.41, 0.16)	−0.44 (−1.27, 0.38)	−0.50 (−1.33, 0.33)	NA	−0.87 (−1.57, −0.18) *
DDS—Regimen-Related Distress	−3.40 (−4.43, −2.36) *	−1.99 (−3.00, −0.97) *	−2.44 (−3.51, −1.37) *	−0.22 (−1.19, 0.75)	−3.18 (−4.60, −1.76) *	−1.77 (−3.17, −0.36) *	2.73 (0.68, 4.77) *	−0.22 (−1.19, 0.75)
DDS—Interpersonal Distress	−0.49 (−0.99, 0.02)	−0.99 (−1.48, −0.49) *	−0.98 (−1.51, −0.46) *	−0.11 (−0.59, 0.37)	−0.20 (−0.70, 0.31)	−0.70 (−1.20, −0.20) *	NA	−0.19 (−0.61, 0.23)
Diabetes Management Self-Efficacy Scale (DSMES)	0.10 (0.06, 0.14) *	0.06 (0.02, 0.10) *	0.11 (0.07, 0.16) *	0.05 (0.01, 0.09) *	0.05 (0.01, 0.09) *	0.01 (−0.03, 0.05)	NA	0.05 (0.02, 0.09) *
DSMES—Healthy Diet	0.11 (0.06, 0.16) *	0.07 (−0.02, 0.12)	0.11 (0.06, 0.15) *	0.03 (−0.01, 0.08)	0.06 (0.01, 0.11) *	0.01 (−0.03, 0.06)	NA	0.04 (−0.01, 0.08)
DSMES—Diet and Blood Sugar	0.11 (0.07, 0.14) *	0.06 (0.02, 0.10) *	**0.08 (0.04, 0.12)**	0.04 (0.01, 0.07) *	0.05 (0.01, 0.08) *	−0.01 (−0.04, 0.03)	NA	0.05 (0.02, 0.08) *
DSMES—Healthy Behaviours	0.09 (0.05, 0.13) *	0.07 (0.03, 0.11) *	0.10 (0.06, 0.14) *	0.07 (0.03, 0.10) *	0.03 (−0.01, 0.07)	0.01 (−0.03, 0.05)	NA	0.06 (0.03, 0.10) *
DSMES—Drug Adherence	0.07 (0.02, 0.11) *	0.05 (−0.00, 0.10)	0.08 (0.04, 0.13) *	0.03 (−0.02, 0.07)	0.04 (0.01, 0.09) *	0.02 (−0.03, 0.07)	NA	0.03 (−0.01, 0.07)

NE: nudging education; NT: nudging tableware; CE: control education; CT: control tableware. All results were adjusted for variables including age, sex, education, family income, and baseline. * for *p* < 0.05 with a bold font; †, independent effect of nudging interventions over time, reflecting the changes in difference over time between nudging education or nudging tableware and time; ‡, the main effect of time, reflecting the changes in the full-control condition over time.

## Data Availability

The data that supporting the findings of this study are available on request from the corresponding author.

## Supplementary Materials

The following supporting information can be downloaded at: https://www.mdpi.com/article/10.3390/nu17091574/s1, Table S1: The details of our nudging education (intervention) and its comparisons with traditional education (control); Table S2: Model Estimation of Intervention Effects by Time (3 months-baseline); Table S3: Estimated Means for outcomes of Each Group on Baseline, 3 months (T1) and 6 months (T2); Table S4: The pairwise comparisons by time points for each group
