# Supplementary material for: Making Diet Management Easier: The Effects of Nudge-Based Dietary Education and Tableware in Individuals with Both T2DM and Overweight/Obesity: A 2 × 2 Cluster Randomized Controlled Trial
Source: Nutrients. 2025 May 3;17(9):1574. doi: 10.3390/nu17091574 (PMC12073172; doi:10.3390/nu17091574)
Supplement: Supplementary file 1 [file nutrients-17-01574-s001.zip › nutrients-3606974-supplementary.pdf]

Contents Page

Table 1 The details of our nudging education (intervention) and its comparisons with traditional education(control)........ 2
Table 2 Model Estimation of Intervention Effects by Time(3 months – baseline) 9
Table 3 Estimated Means for outcomes of Each Group on Baseline, 3 months(T1) and 6 months(T2)........ 11
Table 4 The pairwise comparisons by time points for each group 15

Table S1 The details of our nudging education (intervention) and its comparisons with traditional education(control)

Main Educational Contents	Nudge Strategies	Intervention Practice	Teaching Material Examples	Control Practice
Understanding carbohydrates, the effects of carbohydrates and insulin on blood glucose	Simplification<br>Salience	1. Give practical pictorial examples of carbohydrate-rich foods, simply with two types: staple food (starch) and sugar.<br>2. Use analog legend to explain the digestive process of carbohydrates: brown intestinal tract, red vessels, blue “factory" for pancreas, blue “key” for insulin, yellow “door” for cells, and “keyhole” for insulin receptor.<br>2. A single graph conveys only one piece of information: “Our cells need glucose” and “insulin decreases glucose.”<br>3. Explain that insulin is like a “key” inserted into the keyhole and opens the cell. Once the cell opens, sugar enters the cell, and glucose drops.	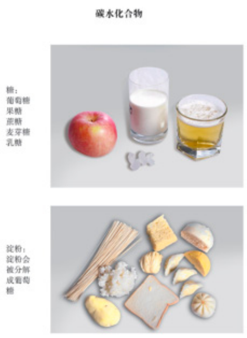<br>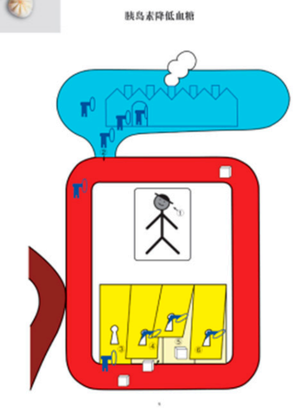	1. Explain the metabolic pathways of blood sugar, including synthesis and decomposition<br>2. Explain the concept of insulin and its function in such metabolic pathways.

Effects of weight on glycemia change	Simplification<br>Priming<br>Salience<br>Ego<br>Affect<br>Pre-commitment	1. Use a “stick man” diagram representing the hypothetical physical condition (normal or overweight).<br>2. Use facial expressions on the “stick man” to prime if such a condition benefits glycemic control.<br>3. Explain that overweight will alert the “keyhole”, thus increasing the demand for “keys” to “open the doors” and the “factory” will be exhausted.<br>4. Explain that reducing weight will recover the “keyhole” and thus regain the ability to decrease glucose as normal people and ease the burden of “factory”.<br>5. Lead them to agree on the importance of losing weight.<br>6. After discussion between the patient and the intervener, determine their weight loss goals and publicly commit themselves.	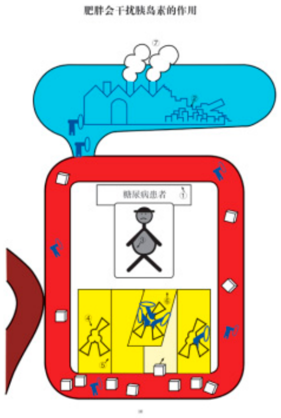<br>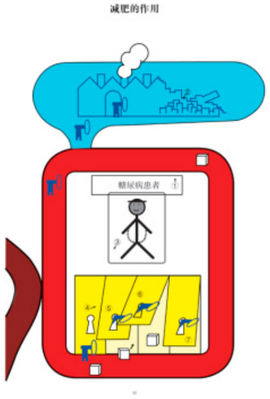	1. Introduce the concept of insulin resistance and explain how and why insulin resistance happens.<br>2. Enlist the harm of insulin resistance<br>3. Explain the concept of “relative insulin deficiency”<br>4. Explain how the body weight affects insulin resistance.

Learning about different types of foods and their nutritional content	Simplification<br>Position<br>Availability	1. Divide foods into three groups and rank them in order of calories: Food rich in water (all non-starchy vegetables, which are considered to have no calories); Food rich in starch and proteins (which are considered to have moderate calories); and food rich in lipid and alcohol which are considered to have high calorie).<br>2. Use food cards as large as physical items (except for vegetables, each food card has a calorie count of 100 kcal) to help patients understand and compare the calorie content of different food categories, as well as the same food with different cooking methods (potato and chips).<br>3. Classify food cards through an interactive game and categorize foods rich in lipids, proteins, carbohydrates, and water into "red, yellow, and green" categories, as well as high-calorie, moderate-calorie, and low-calorie.	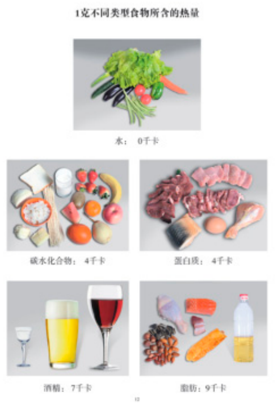<br>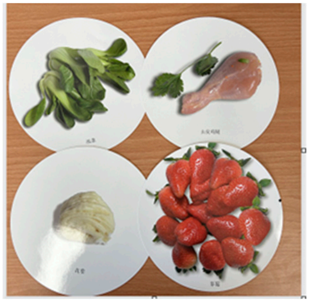<br>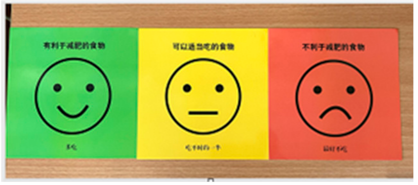	1. Teach them three macronutrients and specific food nutrient information.<br>2. Teach them to recognize and calculate food nutrients and calories with food labels and food composition tables.

Learning about how to engage in a healthy diet	Simplification<br>Priming<br>Position<br>Affect<br>Availability<br>Salience<br>Warning	1. When conducting the game, place green-labeled foods closest to the patient<br>2. Tell them a red card with a crying face means “foods that are not conducive to weight loss—best not to eat,” a yellow card with a regular face means “foods that can be eaten appropriately—only eat half of their usual amount,” and a green card with a smiling face means “foods that are conducive to weight loss—eat more,” consistent with “traffic light.”<br>3. Summarize the three golden diet management rules for weight loss and ask patients to retell them together, using the salient yellow and bold large font and red “lighting” warning signs with corresponding food pictures.	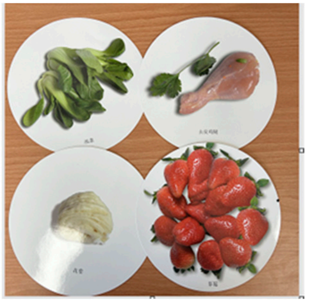<br>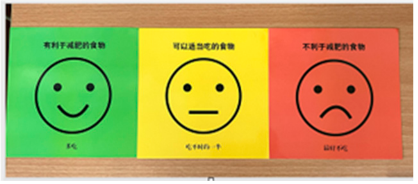<br><br>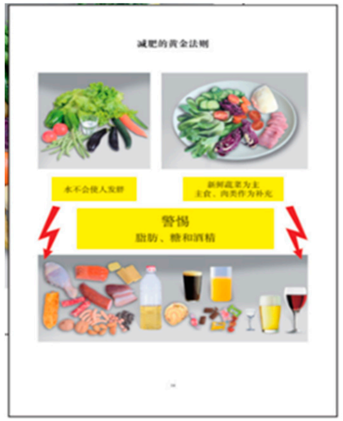 	Following are the three steps: i) Calculate the standard and ideal weight of patients using BMI to determine body shape; 2) Calculate their total daily calorie requirements by their physical activity and body weight according to the reasonable allocation of the three major nutrients; 3)Calculate the weight to be consumed for the categories of food: grains, potatoes, vegetables, fruits, meat, eggs, milk,

				beans, and fats. Use standard tableware, food measuring tools, and food scales to grasp the weight of food accurately.

Individualized instructions	Information<br>Simplification<br>Availability<br>Affect	1. Acknowledge small positive changes in the individual in time;<br>2. Use a visualized "substitution rule" instead of precise calculation, which contains three steps: pick the food cards according to their whole-day meal, replace the higher-calorie food card with a lower-calorie food card until satisfied, and then place them following the classified illustration above, tell them to replace them in daily life.	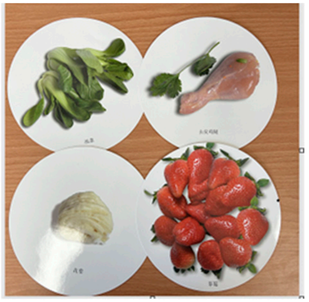<br>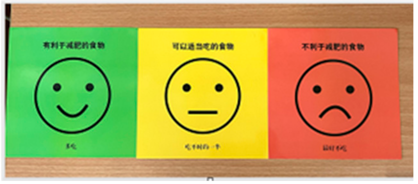	Adjust the diet plan of an individual with precise calculations as introduced above.
Auxiliary materials	Simplification,<br>Reminder<br>Default option	1. Issue a diabetes diary with a given content frame to remind patients to conduct self-monitoring at home every day and give personalized feedback to patients after the next class.<br>2. The patient completes the review questions after class.<br>3. Give them a low literacy and picture-based patient book.	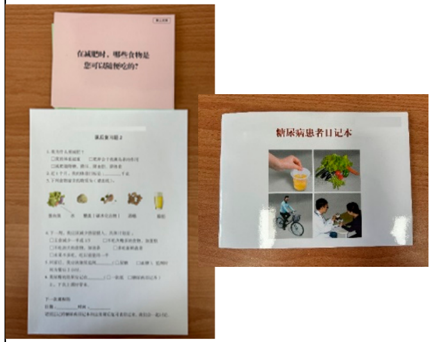	NA

Other processing of the program	Messenger,<br>Social Norm,<br>Ego,<br>Affect,<br>Reminder	1. In the orientation session, the interveners introduce themselves as qualified professionals for this course with a certificate.<br>2. Before each session, measure the weight, blood glucose, and blood pressure of the patient and record them in the diabetes diary to provide feedback.<br>3. Encourage group members to express their management experiences, listen to their demands, and acknowledge good behavior.<br>4. Convey a confidential expectation for their healthy diet management.	NA	NA

Table S2 Model Estimation of Intervention Effects by Time(3 months – baseline)

Outcomes	Dietary Education × Time (NE vs CE)	Tableware × Time (NT vs CT)	Dietary Education × Tableware × Time	Time
	**Adjusted β coefficient (95% CI)**			
Metabolic indicator				
**HbA1C (%)**	**-0.56(-0.85,-0.27)***	**-0.34(-0.63,-0.05)***	NA	-0.03(-0.27,0.21)
**FBG (mmol/L)**	**-0.52(-1.00,-0.03)***	**-0.79(-1.27,-0.31)***	NA	0.04(-0.37,0.45)
**TC (mmol/L)**	**-0.49(-0.84,0.14)***	**-0.69(-1.04,-0.34)***	**0.70(0.19,1.20)***	**0.29(0.05,0.53)***
**TG (mmol/L)**	0.33(-0.08,0.73)	0.13(-0.32,0.57)	NA	-0.19(-0.53,0.15)
**LDL (mmol/L)**	-0.03(-0.22,0.17)	**-0.31(-0.51,-0.12)**	NA	0.05(-0.11,0.22)
**HDL (mmol/L)**	-0.03(-0.18,0.12)	0.09(-0.24,0.56)	NA	0.12(-0.01,0.25)
**BMI (kg/m²)**	**-0.24(-0.61,-0.12)***	**-0.33(-0.70,-0.03)***	NA	-0.20(-0.50,0.11)
Dietary Behaviour				
**Total Calorie(kcal/d)**	**-327.93(-445.46,210.40)***	**-463.55(-585.50,-341.59)***	**332.96(154.74,511.18)***	**90.51(10.47,170.55)***
**Carbohydrate(g/d)**	**-42.92(-63.27,-22.56)***	**-63.34(-87.69,-40.99)***	**44.32(13,46,75,18)***	9.33(-4.53,23.19)
**Protein(g/d)**	**-4.59(-9.28,-0.10)***	**-9.91(-14.63,-5.19)***	NA	0.50(-3.30,4.29)
**Lipid(g/d)**	**-14.89(-21.42,-8.37)***	**-18.44(-25.21,-11.67)***	**16.22(6.33,26.12)***	**5.48(1.03,9.92)***
**Vegetable Intake(g/d)**	**116.34(84.08,148.59)***	**120.88(87.42,154.35)***	**-84.40(-133.31,-35.49)***	-4.40(-26.36,17.51)
Psychological Health				
**Diabetes Distress Scale(DDS)**	**-3.20(-6.01,0.39)***	-1.12(-3.93,1.69)	NA	-0.97(-3.33,1.38)
**DDS-Emotional Burden**	**-2.09(-3.42,-0.76)***	-0.72(-2.03,0.60)	1.85(-0.06,3.77)	0.09(-0.82,1.00)
**DDS-Physician-related Distress**	0.01(-0.89,0.91)	-0.74(-1.64,0.16)	NA	-0.32(-1.07,0.44)
**DDS-Regimen-related Distress**	**-3.02(-4.39,-1.66)***	**-1.38(-2.73,-0.03)***	**2.94(-0.97,4.90)***	0.16(-0.78,1.09)
**DDS-Interpersonal Distress**	-0.41(-0.96,0.15)	-0.54(-1.09,0.02)	NA	0.18(-0.28,0.65)

**Diabetes Management Self-Efficacy Scale (DSMES)**	**0.09(0.04,0.13)***	0.03(-0.01,0.08)	NA	0.02(-0.02,0.06)
**DSMES-Healthy Diet**	**0.09(0.04,0.13)***	0.02(-0.03,0.07)	NA	0.02(-0.02,0.06)
**DSMES-Diet and Blood Sugar**	**0.07(0.03,0.11)***	0.00(-0.04,0.04)	NA	**0.04(0.01,0.07)***
**DSMES-Healthy Behaviours**	**0.06(0.02,0.10)***	0.02(-0.02,0.06)	NA	0.02(-0.01,0.06)*
**DSMES-Drug Adherence**	**0.07(0.02,0.12)***	0.02(-0.02,0.07)	NA	0.01(-0.03,0.05)

Table S3 Estimated Means for outcomes of Each Group on Baseline, 3 months(T1) and 6 months(T2).

Outcomes	Nudging Education Group	Nudging Tableware Group	Combined Group	Full-control Group
	**mean (95% CI)**			
Metabolic indicator				
**HbA1C (%)**				
Baseline	7.55(7.10,8.00)	7.45(7.02,7.89)	7.70(7.25,8.14)	7.63(7.21,8.06)
T1	6.93(6.51,7.35)	7.05(6.65,7.45)	6.80(6.39,7.21)	7.64(7.25,8.03)
T2	6.51(6.10,7.35)	6.86(6.47,7.24)	6.69(6.31,7.07)	7.70(7.32,8.07)
**FBG (mmol/L)**				
Baseline	8.40(7.59,9.14)	8.76(7.77,9.52)	8.41(7.70,9.17)	8.13(7.41,8.86)
T1	7.76(7.01,8.51)	7.92(7.20,8.63)	7.27(6.55,7.99)	8.27(7.57,8.98)
T2	7.43(6.70,8.17)	7.50(6.80,8.20)	7.13(6.42,7.83)	8.40(7.70,9.08)
**TC (mmol/L)**				
Baseline	4.67(4.36,4.98)	4.95(4.66,5.25)	4.78(4.47,5.09)	4.54(4.25,4.83)
T1	4.47(4.16,4.78)	4.55(4.25,4.85)	4.59(4.28,4.89)	4.82(4.53,5.11)
T2	4.34(4.03,4.65)	4.64(4.34,4.94)	4.38(4.07,4.69)	4.91(4.62,5.21)
**TG (mmol/L)**				
Baseline	1.70(1.28,2.12)	2.52(2.11,2.93)	1.94(1.51,2.37)	2.03(1.52,2.32)
T1	1.62(1.28,1.97)	2.00(1.66,2.34)	1.91(1.84,2.54)	1.96(1.60,2.23)
T2	2.16(1.72,2.59)	1.97(1.54,2.40)	1.87(1.42,2.31)	2.16(1.74,2.60)
**LDL (mmol/L)**				
Baseline	2.62(2.30,3.01)	3.31(3.00,3.62)	3.12(2.80,3.43)	3.14(2.83,3.43)
T1	2.55(2.24,2.87)	2.96(2.65,3.27)	2.94(2.63,3.25)	3.28(2.97,3.58)

T2	2.60(2.28,3.00)	3.03(2.72,3.31)	2.77(2.46,3.08)	3.24(2.94,3.55)
**HDL (mmol/L)**				
Baseline	1.25(1.07,1.43)	1.28(1.10,1.45)	1.20(1.02,1.34)	1.17(1.00,1.35)
T1	1.36(1.20,1.52)	1.32(1.16,1.47)	1.18(1.02,1.34)	1.29(1.13,1.44)
T2	1.20(1.01,1.40)	1.27(1.10,1.45)	1.20(0.98,1.38)	1.46(1.27,1.64)
**BMI (kg/m^2)**				
Baseline	27.48(26.27,28.69)	27.63(26.79,29.12)	27.57(26.39,28.54)	27.20(26.05,28.36)
T1	26.99(25.78,28.21)	27.38(26.21,28.56)	26.85(25.66,28.03)	27.05(25.89,28.21)
T2	26.98(25.76,28.18)	27.16(25.98,28.35)	26.76(25.57,27.96)	27.28(26.11,28.45)
Dietary Behaviour				
**Total Calorie(kcal/d)**				
Baseline	1705.85(1575.89,1835.81)	1621.76(1486.77,1756.75)	1715.12(1577.98,1852.27)	1606.39(1479.39,1733.39)
T1	1468.43(1351.50,1585.36)	1248.72(1128,37,1369.08)	1347.12(1227.27,1466.97)	1696.90(1579.94.1813.86)
T2	1532.77(1413.02,1652.51)	1285.99(1162.46,1409.52)	1360.89(1237.21,1484.58)	1725.22(1606.19,1844.25)
**Carbohydrate(g/d)**				
Baseline	224.99(204.80,245.17)	211.12(190.20,232.03)	222.15(200.96,243.34)	212.25(192.49,232.01)
T1	191.40(172.59,210.21)	157.20(137.82,176.58)	169.63(150.27,188.99)	221.58(202.89,240,27)
T2	199.59(180.39,219.78)	166.61(146.81,186.41)	165.41(145.53,185.29)	232.16(213.18,251.14)
**Protein(g/d)**				
Baseline	68.11(63.65,72.57)	62.55(57.77,67.33)	66.65(61.62,71.68)	64.61(60.43,68.78)
T1	63.47(60.02,66.92)	52.51(48.82,56.21)	53.35(49.47,57.22)	65.58(62.34,68.82)
T2	65.22(61.77,68.66)	53.31(29.61,57.01)	57.05(53.17,60.92)	64.16(60.92,67.40)
**Lipid(g/d)**				
Baseline	49.95(42.48,57.43)	50.17(42.31,58.02)	53.85(46.02,61.69)	48.71(41.20,56.22)
T1	40.54(33.76,47.32)	37.21(30.09,44.32)	42.22(35.30,49.14)	54.19(47.20,61.18)
T2	43.28(36.33,50.22)	36.78(29.49,44.07)	43.98(36.83,51.13)	53.26(46.15,60.37)

**Vegetable Intake(g/d)**				
Baseline	217.04(167.32,266.75)	216.23(162.35,270.12)	233.94(183.41,284.47)	224.88(171.54,278.21)
T1	328.98(278.46,379.49)	332.72(278.10,287.34)	382.36(330.78,433.94)	220.48(166.65,274.31)
T2	332.25(282.30,382.20)	328.41(274.32,382.50)	380.35(329,50,431.19)	217.85(164.39,271.31)
Psychological Health				
**Diabetes Distress Scale(DDS)**				
Baseline	31.78(28.10,35.45)	29.61(26.04,33.18)	30.71(27.05,34.38)	30.25(26.76,33.74)
T1	26.15(22.92,29.39)	28.12(24.99,31.25)	26.97(23.79,30.15)	30.56(27.48,33.65)
T2	23.73(20.73,26.73)	25.51(22.61,28.41)	23.90(20.99,26.81)	29.17(26.29,32.05)
**DDS-Emotional Burden**				
Baseline	9.81(8.57,11.05)	8.35(7.15,9.55)	8.86(7.62,10.09)	9.06(7.88,10.24)
T1	7.81(6.66,8.95)	7.72(6.61,8.83)	7.99(6.87,9.13)	9.15(8.05,10.25)
T2	7.25(6.17,8.33)	6.85(5.80,7.89)	6.98(5.92,8.04)	8.93(7.89,9.97)
**DDS-Physician-related Distress**				
Baseline	6.49(5.61,7.38)	6.74(5.88,7.61)	6.77(5.86,7.67)	6.86(6.03,7.70)
T1	6.03(5.27,6.79)	5.53(4.79,6.27)	5.88(5.10,6.65)	6.68(5.96,7.40)
T2	4.89(4.31,5.47)	5.10(4.54,5.66)	5.25(4.67,5.83)	6.24(5.69,6.79)
**DDS-Regimen-related Distress**				
Baseline	11.45(9.97,12.93)	9.89(8.45,11.33)	10.27(8.80,11.74)	9.80(8.39,11.22)
T1	8.58(7.22,9.95)	8.66(7.34,9.99)	8.96(7.61,10.30)	9.96(8.65,11.26)
T2	8.05(6.76,9.35)	7.90(6.64,9.16)	7.83(6.56,9.10)	9.58(8.34,10.82)
**DDS-Interpersonal Distress**				
Baseline	4.11(3.49,4.74)	4.69(4.08,5.30)	4.87(4.23,5.50)	4.63(4.04,5.22)
T1	3.82(3.29,4.35)	4.26(3.75,4.78)	4.19(3.65,4.72)	4.88(4.38,5.38)
T2	3.63(3.19,4.07)	3.70(3.27,4.13)	3.88(3.45,4.32)	4.52(4.11,4.94)

**Diabetes Management Self-Efficacy Scale (DSMES)**				
Baseline	0.75(0.70,0.80)	0.77(0.72,0.81)	0.76(0.71,0.81)	0.74(0.70,0.79)
T1	0.85(0.80,0.89)	0.81(0.77,0.86)	0.90(0.85,0.95)	0.77(0.72,0.81)
T2	0.87(0.83,0.92)	0.83(0.79,0.87)	0.87(0.83,0.91)	0.80(0.76,0.84)
**DSMES-Healthy Diet**				
Baseline	0.70(0.65,0.75)	0.71(0.66,0.76)	0.70(0.65,0.75)	0.69(0.65,0.74)
T1	0.82(0.78,0.86)	0.76(0.72,0.80)	0.81(0.77,0.85)	0.71(0.66,0.75)
T2	0.81(0.77,0.85)	0.75(0.70,0.79)	0.80(0.76,0.85)	0.73(0.69,0.77)
**DSMES-Diet and Blood Sugar**				
Baseline	0.76(0.70,0.81)	0.79(0.73,0.84)	0.79(0.73,0.85)	0.78(0.73,0.83)
T1	0.87(0.82,0.92)	0.83(0.78,0.88)	0.89(0.84,0.94)	0.81(0.76,0.86)
T2	0.86(0.81,0.91)	0.84(0.80,0.89)	0.87(0.82,0.92)	0.82(0.77,0.86)
**DSMES-Healthy Behaviours**				
Baseline	0.76(0.71,0.81)	0.75(0.71,0.80)	0.77(0.72,0.82)	0.75(0.70,0.80)
T1	0.84(0.80,0.88)	0.79(0.79,0.80)	0.87(0.83,0.91)	0.76(0.72,0.80)
T2	0.84(0.80,0.87)	0.82(0.78,0.86)	0.86(0.83,0.90)	0.80(0.76,0.84)
**DSMES-Drug Adherence**				
Baseline	0.84(0.78,0.89)	0.84(0.78,0.89)	0.79(0.74,0.85)	0.80(0.74,0.85)
T1	0.92(0.87,0.97)	0.87(0.82,0.93)	0.90(0.84,0.95)	0.81(0.76,0.86)
T2	0.90(0.85,0.96)	0.89(0.94,0.94)	0.88(0.83,0.92)	0.82(0.77,0.87)

Table S4 The pairwise comparisons by time points for each group

Outcomes	T1-T0		T2-T0		T2-T1	
	Mean difference	P	Mean difference	P	Mean difference	P
Metabolic indicator						
**Hb1AC(%)**						
Nudging Education Group	**-0.62(-0.91,-0.33)**	**＜0.001**	**-1.01(-1.33,-0.68)***	**＜0.001**	**-0.42(-0.66,-0.20)***	**＜0.001**
Nudging Tableware Group	**-0.40(-0.69,-0.12)***	**0.006**	**-0.60(-0.91,-0.29)***	**＜0.001**	-0.19(-0.42,-0.03)	0.094
Combined Group	**-0.89(-1.20,-0.60)***	**＜0.001**	**-1.04(-1.36,-0.73)***	**＜0.001**	-0.11(-0.34,0.13)	0.366
Full-Control Group	0.01(-0.27,-0.28)	0.995	0.06(-0.24,0.36)	0.711	0.06(-0.16,0.27)	0.618
**FBG(mmol/L)**						
Nudging Education Group	**-0.60(-1.09,-0.10)***	**0.018**	**-0.92(-1.47,-0.38)***	**0.001**	-0.33(-0.78,0.13)	0.156
Nudging Tableware Group	**-0.86(-1.35,-0.38)***	**＜0.001**	**-1.27(-1.81,-0.75)***	**＜0.001**	-0.42(-0.86,0.03)	0.066
Combined Group	**-1.14(-1.65,-0.64)***	**＜0.001**	**-1.29(-1.85,-0.73)***	**＜0.001**	-0.14(-0.60,0.33)	0.548
Full-Control Group	0.14(-0.32,0.61)	0.543	0.26(-0.25,0.77)	0.332	0.12(-0.31,0.54)	0.596
**TC(mmol/L)**						
Nudging Education Group	-0.20(-0.46,0.05)	0.121	**-0.33(-0.63,-0.04)**	**0.025**	-0.13(-0.37,0.11)	0.281
Nudging Tableware Group	**-0.40(-0.65,-0.15)***	**0.002**	**-0.32(-0.60,-0.03)***	**0.03**	0.09(-0.15,0.32)	0.473
Combined Group	-0.20(-0.46,0.07)	0.15	**-0.40(-0.70,-0.10)***	**0.01**	-0.21(0.10,-0.45)	0.101
Full-Control Group	**0.29(0.05,0.53)***	**0.02**	**0.38(0.10,0.65)***	**0.007**	0.09(-0.13,0.31)	0.422
**TG(mmol/L)**						
Nudging Education Group	-0.08(-0.48,0.33)	0.714	0.46(-0.06,0.97)	0.083	**0.53(0.16,0.90)***	**0.005**
Nudging Tableware Group	**-0.51(-0.91,-0.11)***	**0.012**	**-0.55(-1.05,-0.04)***	**0.033**	-0.03(-0.40,0.33)	0.855
Combined Group	0.24(-0.18,0.66)	0.254	-0.07(-0.60,0.46)	0.787	-0.32(-0.70,0.06)	0.102
Full-Control Group	-0.08(-0.38,0.27)	0.481	0.23(-0.25,0.72)	0.344	0.31(-0.11,0.58)	0.187
**LDL(mmol/L)**						
Nudging Education Group	-0.07(-0.27,0.13)	0.48	-0.03(-0.25,0.20)	0.819	0.05(-0.14,0.23)	0.634

Nudging Tableware Group	**-0.35(-0.55,-0.16)***	**< 0.001**	**-0.28(-0.50,-0.07)***	**0.011**	0.07(-0.11,0.25)	0.448
Combined Group	**-0.38(-0.58,-0.13)***	**0.039**	**-0.35(-0.58,-0.12)***	**0.003**	-0.17(-0.36,0.02)	0.075
Full-Control Group	0.14(-0.04,0.33)	0.133	0.11(-0.10,0.32)	0.304	-0.03(-0.21,0.14)	0.706
**HDL(mmol/L)**						
Nudging Education Group	0.11(-0.04,0.26)	0.158	-0.05(-0.24,0.15)	0.635	-0.05(-0.24,0.15)	0.635
Nudging Tableware Group	0.04(-0.11,0.19)	0.58	-0.01(-0.20,0.18)	0.919	-0.05(-0.22,0.12)	0.554
Combined Group	-0.01(-0.17,0.14)	0.858	-0.01(-0.21,0.19)	0.899	0.01(-0.16,0.18)	0.965
Full-Control Group	0.11(-0.03,0.25)	0.131	**0.28(0.10,0.47)***	**0.003**	**0.17(0.01,0.34)***	**0.041**
**BMI(Kg/m2)**						
Nudging Education Group	**-0.49(-0.86,-0.12)***	**0.01**	**-0.50(-0.89,-0.10)***	**0.015**	-0.01(-0.27,0.26)	0.957
Nudging Tableware Group	**-0.57(-0.94,-0.21)***	**0.002**	**-0.79(-1.18,-0.40)***	**< 0.001**	-0.22(-0.48,0.04)	0.097
Combined Group	**-0.72(-1.10,-0.34)***	**< 0.001**	**-0.80(-0.12,-0.39)**	**< 0.001**	-0.09(-0.36,0.19)	0.543
Full-Control Group	-0.15(-0.50,0.20)	0.389	0.08(-0.20,0.45)	0.681	0.23(-0.02,0.48)	0.069
Dietary Behaviour						
**Total Calorie Intake(kcal/d)**						
Nudging Education Group	**-237.42 (-323.48, -151.35)***	**< 0.001**	**-173.08 (-265.00, -81.16)***	**< 0.001**	64.34(-7.64,136.32)	0.08
Nudging Tableware Group	**-373.04 (-465.05, -281.03)***	**< 0.001**	**-335.77 (-434.04, -237.50)***	**< 0.001**	37.27 (-39.68,144.22)	0.341
Combined Group	**-368.01 (-465.38, -270.63)***	**< 0.001**	**-354.23 (-458.23, -250.23)***	**< 0.001**	13.78(-67.66,95.21)	0.739
Full-Control Group	**90.51(10.47,170.55)***	**0.027**	**118.82(33.34,204.31)***	**0.007**	28.31(-38.63,95.26)	0.406
**Carbohydrate Intake (g/d)**						
Nudging Education Group	**-33.59(-48.49,-18.68)***	**< 0.001**	**-25.40(-40.17,10.64)***	**0.001**	8.19(-4.66,21.040	0.211
Nudging Tableware Group	**-53.92(-69.85,-37.98)***	**< 0.001**	**-44.51(-60.29,-28.72)***	**< 0.001**	9.41(-4.33,23.15)	0.179
Combined Group	**-52.52(-69.38,-35.65)***	**< 0.001**	**-56.74(-73.44,-40.03)***	**< 0.001**	-4.22(-18.76,10.32)	0.568

Full-Control Group	9.33(-4.53,23.19)	0.186	**19.91(6.18,33.64)***	**0.005**	10.58(-1.37,22.53)	0.082
**Protein Intake (g/d)**						
Nudging Education Group	**-4.64(-9.21,-0.07)***	**0.047**	-2.90(-7.88,2.09)	0.253	1.74(-2.19,5.68)	0.384
Nudging Tableware Group	**-10.04(-14.93,-5.15)***	**< 0.001**	**-9.24(-14.57,-3.91)***	**0.001**	0.80(-3.41,5.01)	0.709
Combined Group	**-13.30(-18.48,-8.13)***	**< 0.001**	**-9.61(-15.24,-3.97)***	**< 0.001**	3.70(-0.75,8.15)	0.103
Full-Control Group	0.97(-3.28,5.23)	0.653	-0.44(-5.01,4.19)	0.851	-1.42(-5.08,2.25)	0.447
**Lipid Intake (g/d)**						
Nudging Education Group	**-9.42(-14.19,-4.64)***	**< 0.001**	**-6.68(-11.60,-1.76)***	**0.008**	2.74(-1.20,6.67)	0.172
Nudging Tableware Group	**-12.96(-19.07,-7.86)***	**< 0.001**	**-13.39(-18.65,-8.13)***	**< 0.001**	-0.3(-4.64,3.78)	0.841
Combined Group	**-11.64(-18.04,6.23)***	**< 0.001**	**-9.87(-15.44,-4.31)***	**0.001**	1.76(-2.69,6.22)	0.436
Full-Control Group	**5.48(1.03,9.92)***	**0.016**	**4.55(0.03,9.13)***	**0.038**	-0.93(-4.59,2.73)	0.618
**Vegetable Intake(g/d)**						
Nudging Education Group	**111.94(88.32,135.56)***	**< 0.001**	**115.21(91.15,139.28)***	**< 0.001**	3.27(-20.64,27.19)	0.788
Nudging Tableware Group	**116.49(91.24,141.74)***	**< 0.001**	**112.17(86.44,137.90)***	**< 0.001**	4.31(-21.25,29.88)	0.74
Combined Group	**148.42(121.70,175.15)***	**< 0.001**	**146.41(119.17,173.64)***	**< 0.001**	-2.02(-29.07,25.04)	0.883
Full-Control Group	-4.40(-26.36,17.57)	0.694	-7.03(-29.41,15.36)	0.537	-2.63(-24.87,19.61)	0.816
Psychological Status						
**Diabetes Distress Scale (DDS) Total Score**						
Nudging Education Group	**-3.63(-6.46,-0.80)***	**0.015**	**-6.05(-8.81,-5.28)***	**< 0.001**	**-2.42(-4.26,-0.58)***	**0.01**
Nudging Tableware Group	-2.08(-6.25,0.71)	0.074	**-3.09(-5.80,-0.40)***	**0.021**	**-2.61(-4.42,-0.81)***	**0.005**
Combined Group	**-3.74(-6.66,-0.83)***	**0.012**	**-6.81(-9.66,-3.97)***	**< 0.001**	**-3.07(-4.97,1.17)***	**0.002**
Full-Control Group	0.31(-2.35,2.97)	0.818	**-2.80(-3.68,-1.51)***	**0.004**	**-1.39(-3.13,-0.34)***	**0.015**
**DDS-emotional burden**						
Nudging Education Group	**-2.00(-2.97,-1.03)***	**< 0.001**	**-2.56(-3.45,-1.67)***	**< 0.001**	-0.56(-1.25,0.13)	0.111
Nudging Tableware Group	-0.63(-1.57,0.32)	0.194	**-1.50(-2.37,-0.63)***	**0.001**	**-0.88(-1.55,-0.21)***	**0.011**
Combined Group	**-1.00(-1.86,-0.14)***	**0.039**	**-1.88(-2.80,-0.95)***	**< 0.001**	**-1.02(-1.73,-0.31)***	**0.005**

Full-Control Group	0.09(-0.82,1.00)	0.845	-0.13(-0.897,0.71)	0.761	-0.22(-0.87,0.43)	0.503
**DDS-physician-related distress**						
Nudging Education Group	-0.46(-1.38,0.45)	0.319	**-1.60(-2.44,-0.77)***	**< 0.001**	**-1.14(-1.77,-0.51)***	**< 0.001**
Nudging Tableware Group	**-1.21(-2.10,0.32)***	**0.008**	**-1.65(-2.47,-0.83)***	**< 0.001**	-0.44(-1.06,0.19)	0.168
Combined Group	-0.89(-1.83,0.05)	0.063	**-1.52(-2.38,-0.65)***	**0.001**	-0.63(-1.28,0.03)	0.061
Full-Control Group	-0.18(-1.04,0.68)	0.678	-0.62(-1.41,0.16)	0.146	-0.44(-1.04,0.15)	0.146
**DDS-regimen-related distress**						
Nudging Education Group	**-2.87(-3.86,-1.87)***	**< 0.001**	**-3.40(-4.43,-2.36)***	**< 0.001**	-0.53(-1.29,0.23)	0.172
Nudging Tableware Group	**-1.23(-2.20,-0.25)***	**0.014**	**-1.99(-3.00,-0.97)***	**< 0.001**	**-0.76(-1.50,-0.02)***	**0.045**
Combined Group	**-1.31(-2.34,-0.29)***	**0.012**	**-2.44(-3.51,-1.37)***	**< 0.001**	**-1.13(-1.91,-0.34)***	**0.005**
Full-Control Group	0.16(-0.78,1.09)	0.744	-0.22(-1.19,0.75)	0.656	-0.38(-1.09,0.34)	0.301
**DDS-interpersonal distress**						
Nudging Education Group	-0.29(-0.86,0.27)	0.305	-0.49(-0.99,0.02)	0.062	-0.19(-0.54,0.16)	0.288
Nudging Tableware Group	-0.42(-0.97,0.13)	0.133	**-0.99(-1.48,-0.49)***	**< 0.001**	**-0.56(-0.91,-0.22)***	**0.001**
Combined Group	**-0.68(-1.26,-0.10)***	**0.021**	**-0.98(-1.51,-0.46)***	**< 0.001**	-0.30(-0.67,0.06)	0.102
Full-Control Group	0.25(-0.28,0.78)	0.36	-0.11(-0.59,0.37)	0.661	**-0.35(-0.69,-0.02)***	**0.036**
**Diabetes Management Self-efficacy Scale(DSMES) Total Score**						
Nudging Education Group	**0.10(0.05,0.15)***	**< 0.001**	**0.10(0.06,0.14)***	**< 0.001**	0.01(-0.03,0.04)	0.884
Nudging Tableware Group	0.05(-0.00,0.09)	0.059	**0.06(0.02,0.10)***	**0.004**	0.02(-0.02,0.06)	0.419
Combined Group	**0.14(0.09,0.19)***	**< 0.001**	**0.11(0.07,0.16)***	**< 0.001**	-0.03(-0.07,0.01)	0.173
Full-Control Group	0.02(-0.02,0.07)	0.343	**0.05(0.01,0.09)***	**0.01**	0.03(-0.00,0.07)	0.074
**DSMES-Healthy Diet**						
Nudging Education Group	**0.12(0.07,0.17)***	**< 0.001**	**0.11(0.06,0.16)***	**< 0.001**	-0.01(-0.05,0.03)	0.656
Nudging Tableware Group	0.05(-0.01,0.10)	0.073	**0.07(0.02,0.12)***	**0.003**	0.02(-0.02,0.05)	0.327
Combined Group	**0.12(0.06,0.16)***	**< 0.001**	**0.11(0.06,0.15)***	**< 0.001**	-0.01(-0.04,0.03)	0.799
Full-Control Group	0.01(-0.04,0.05)	0.705	0.03(-0.01,0.08)	0.139	0.03(-0.01,0.06)	0.148

**DSMES-Diet and Blood Sugar**						
Nudging Education Group	**0.11(0.07,0.15)***	**< 0.001**	**0.11(0.07,0.14)***	**< 0.001**	-0.01(-0.04,0.02)	0.568
Nudging Tableware Group	**0.05(0.01,0.09)***	**0.019**	**0.06(0.02,0.10)***	**0.002**	0.01(-0.02,0.04)	0.041
Combined Group	0.10(0.06,0.15)*	< 0.001	**0.08(0.04,0.12)**	**< 0.001**	-0.02(-0.05,0.01)	0.12
Full-Control Group	0.04(0.00,0.07)*	0.031	**0.04(0.01,0.07)***	**0.04**	0.02(-0.02,0.06)	0.085
**DSMES-Healthy Behaviours**						
Nudging Education Group	**0.08(0.04,0.13)***	**< 0.001**	**0.09(0.05,0.13)***	**< 0.001**	0.01(-0.03,0.040	0.835
Nudging Tableware Group	0.04(-0.01,0.08)	0.104	**0.07(0.03,0.11)***	**0.002**	0.02(-0.01,0.05)	0.151
Combined Group	**0.10(0.06,0.15)***	**< 0.001**	**0.10(0.06,0.14)***	**< 0.001**	-0.01(-0.04,0.03)	0.729
Full-Control Group	0.02(-0.02,0.06)	0.263	**0.07(0.03,0.10)***	**0.001**	**0.04(0.01,0.07)***	**0.005**
**DSMES-Drug Adherence**						
Nudging Education Group	**0.08(0.04,0.13)***	**< 0.001**	**0.07(0.02,0.11)***	**0.004**	-0.02(-0.05,0.02)	0.372
Nudging Tableware Group	0.04(-0.01,0.09)	0.089	0.05(-0.00,0.10)	0.065	0.01(-0.03,0.05)	0.624
Combined Group	**0.11(0.06,0.15)***	**< 0.001**	**0.08(0.04,0.13)***	**0.001**	-0.02(-0.06,0.02)	0.257
Full-Control Group	0.01(-0.03,0.06)*	0.579	0.03(-0.02,0.07)	0.264	0.01(-0.02,0.05)	0.483

Table S5 Baseline characters of participants in cluster level

Variables	Nudging Education Group (N= 3)	Nudging Tableware Group (N= 3)	Combined Group (N= 3)	Full-Control Group (N=3)
Demographic				
Age (years)	53.35(1.64)	53.45(1.63)	53.28(1.22)	50.29(8.84)
Metabolic indicator				
HbA1C(%)	7.51(0.34)	7.54(0.23)	7.73(0.45)	7.70(0.35)
Fasting blood glocose(FBG,mmol/L))	8.27(0.39)	8.96(0.33)	8.54(1.18)	8.32(1.34)
Total cholesterol(TC,mmol/L)	4.55(0.39)	4.96(0.19)	4.74(0.21)	4.57(0.46)
Triglyceride(TG,mmol/L)	1.68(0.30)	2.68(0.72)	1.86(0.19)	1.99(0.38)
Low density lipoprotein(LDL,mg/dL)	2.64(0.52)	3.31(0.27)	3.14(0.19)	3.13(0.48)
High density lipoprotein(HDL,mg/dL)	1.22(0.05)	1.25(0.20)	1.22(0.03)	1.14(0.17)
Body mass index(BMI, kg/m^2)	27.34(0.94)	28.29(0.62)	27.42(1.03)	27.70(2.00)
**Dietary Behaviour**				
Total Calorie Intake (kcal/d)	1693.97(135.43)	1603.26(54.66)	1703.30(181.90)	1620.74(103.74)
Carbohydrate Intake(g/d)	222.05(27.56)	201.08(6.32)	225.32(24.86)	214.14(2.54)
Protein Intake(g/d)	65.50(8.32)	63.79(5.74)	65.56(7.84)	65.11(6.10)
Lipid Intake(g/d)	52.00(12.27)	51.22(3.96)	51.80(7.20)	48.41(8.64)
Vegetable Intake(g/d)	228.49(70.81)	234.01(36.43)	218.79(22.43)	225.73(14.42)
Psychological status				
Diabetes Distress Score	35.65(10.37)	29.40(5.79)	30.57(7.61)	29.56(3.99)
Diabetes Self-management Efficacy Score	0.73(0.05)	0.77(0.02)	0.76(0.08)	0.75(0.04)
